# Kohonen neural network and symbiotic-organism search algorithm for intrusion detection of network viruses

**DOI:** 10.3389/fncom.2023.1079483

**Published:** 2023-02-22

**Authors:** Guo Zhou, Fahui Miao, Zhonghua Tang, Yongquan Zhou, Qifang Luo

**Affiliations:** ^1^Department of Science and Technology Teaching, China University of Political Science and Law, Beijing, China; ^2^College of Artificial Intelligence, Guangxi University for Nationalities, Nanning, China; ^3^Guangxi Key Laboratories of Hybrid Computation and Integrated Circuit (IC) Design Analysis, Nanning, China

**Keywords:** intrusion detection, symbiotic-organism search algorithm, Kohonen neural network, detection rate, false alarm rate

## Abstract

**Introduction:**

The development of the Internet has made life much more convenient, but forms of network intrusion have become increasingly diversified and the threats to network security are becoming much more serious. Therefore, research into intrusion detection has become very important for network security.

**Methods:**

In this paper, a clustering algorithm based on the symbiotic-organism search (SOS) algorithm and a Kohonen neural network is proposed.

**Results:**

The clustering accuracy of the Kohonen neural network is improved by using the SOS algorithm to optimize the weights in the Kohonen neural network.

**Discussion:**

Our approach was verified with the KDDCUP99 network intrusion data. The experimental results show that SOS-Kohonen can effectively detect intrusion. The detection rate was higher, and the false alarm rate was lower.

## 1. Introduction

With the rapid spread of the Internet, there has also been a rapid development of online systems for shopping, banking, making payments, stock trading, and so on. However, due to the openness of the network, forms of network intrusion are becoming increasingly diversified, so that networks and systems are experiencing ever more serious threats. Therefore, detecting network intrusion has become a critical issue in network security. In recent years, increasing attention has been paid by scholars all over the world to intrusion detection. The aim is to identify any behavior that could compromise the integrity, confidentiality, or availability of the system. It can be defined as identifying the people accessing a computer system (Shitharth and Winston, 2017). Current methods of network intrusion detection can be divided into two categories: misuse intrusion detection and abnormal intrusion detection. The capability of misuse intrusion detection mainly depends on the completeness of the detection knowledge base. Its shortcoming is that it cannot find unknown forms of intrusion. Abnormal intrusion detection is based on identifying a difference between the detected and acceptable behavior.

Due to their continuous development, various swarm intelligence algorithms have been applied to intrusion detection, such as the genetic algorithm (Mabu et al., 2011), immune algorithm (Zhang et al., 2014), ant colony optimization (Feng et al., 2014), and so on. However, as the “no free lunch” theorem (Wolpert and Macready, 1997) argues, none of these group intelligence algorithms is suitable for detecting all forms of intrusion. Thus, finding a better algorithm is still a hot topic for scholars in various countries.

In recent years, various meta-heuristic algorithms have been proposed, such as the crow search algorithm (Askarzadeh, 2016; Hussien et al., 2021), monarch butterfly optimization (Wang et al., 2019), lightning search algorithm (Shareef et al., 2015; Abualigah et al., 2021), water evaporation optimization (Kaveh and Bakhshpoori, 2016), Kohonen neural network (Wehrens and Buydens, 2007; De Almeida et al., 2013), symbiotic-organism search (SOS) algorithm (Cheng and Prayogo, 2014; Ezugwua and Prayogo, 2019; Chakraborty et al., 2022), bat algorithm (BA) (Xinshe, 2010; Shehab et al., 2022), cuckoo algorithm (CS) (Yang and Deb, 2009), flower pollination algorithm (FPA) (Yang, 2012; Fouad and Gao, 2019), grey wolf optimizer (GWO) (Mirjalili et al., 2014), particle swarm algorithm (PSO) (Kennedy and Eberhart, 1995; Jordehi and Jasni, 2015), Harris hawk optimization(Hussien et al., 2022), Quantum-inspired deep neural networks (Shi et al., 2021), Gaining–sharing knowledge based algorithm (Mohamed et al., 2020) and so on.

This paper proposes a clustering algorithm based on the SOS algorithm and a Kohonen neural network. The clustering accuracy of Kohonen neural network was improved by using the SOS algorithm to optimize the weights of the Kohonen neural network. SOS-Kohonen was tested with the KDDCUP99 network intrusion data. The experimental results show that it can effectively detect intrusion detection. Compared to other standard methods, the detection rate was higher and the false alarm rate was lower.

The remainder of the paper is organized as follows. Section 2 describes the structure of a Kohonen neural network. Section 3 introduces a basic SOS algorithm. Section 4 considers the use of the SOS-Kohonen algorithm for intrusion detection. Section 5 describes the data preprocessing method. The simulation experiments and results are presented in Sections 6, 7 concludes and discusses future work.

## 2. Kohonen neural network

**FIGURE 1:**
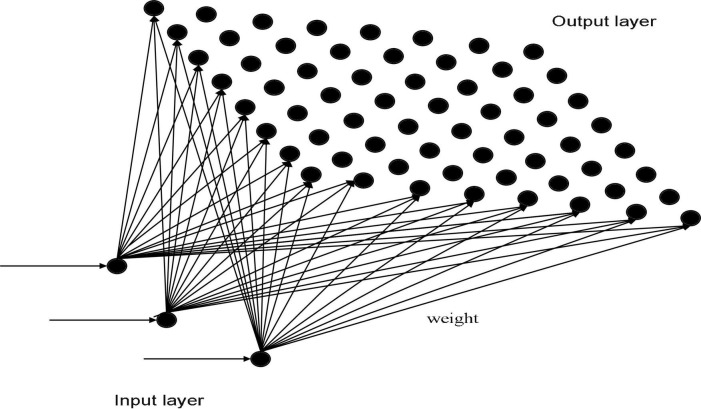
Structure of a Kohonen neural network.

Finnish professor Teuvo Kohonen proposed an unsupervised self-organizing competitive neural network called a Kohonen neural network. It can achieve automatic clustering by using a self-organizing feature mapping to adjust network weights. A Kohonen neural network (De Almeida et al., 2013) consists of two feedforward layers, namely an input layer and an output layer. The input layer is mapped into a two-dimensional response mesh in the output layer based on weights. The topology of a Kohonen neural network is shown in Figure 1.

In a Kohonen neural network, the Euclidean distance of each neuron is obtained by calculating the input eigenvector for the corresponding output layer. The neuron with the smallest Euclidean distance is the superior neuron, and its connection weights are adjusted to make it closer to the original input vector. The area adjacent to the winning neuron is also adjusted by the connection weight to make it closer to the input vector.

In the training phase, each input vector *X*_*s*_ is input into the network, and only those winning neurons closest to the current weight vector of the input receive a corresponding stimulus. The pattern vector *X*_*s*_ is calculated as the minimum Euclidean distance from the selected winning neurons:


(1) $$n\,e\,u\,r\,o\,n\,c\leftarrow \min_{j}\left\{\sum_{i}{\left(x_{s\,i}-w_{j\,i}\right)}^{2}\right\},j=1,2,\ldots ,N\times N$$

where *c* represents the winning neuron and *x*_*si*_ represents the *i*th coordinate of the input vector. In addition, the level of the *i*th weight of neuron *j* is denoted by *w*_*ji*_. The number of neurons in a Kohonen level is denoted by *N*×*N*. Once the winning neuron is selected, the corresponding weight *w*_*ji*_ of each neuron *j* in the layer is updated according to the difference between the original weight and the input neuron, as follows:


(2) $$\Delta \,w_{j\,i}=\eta \,\left(1-\frac{d_{r}}{d_{\max}+1}\right)\,\left(x_{s\,i}-w_{j\,i}^{o\,l\,d}\right),d_{r}=0,1,\ldots ,d_{\max}$$

where the learning rate is η, the weight of the previous generation of *w*_*ji*_ is $w_{j\,i}^{o\,l\,d}$, and the number of neurons between neuron *j* and the superior neuron is represented by the topological distance *d*_*r*_. The size of the adjacent area *d*_*max*_ decreases from the coverage of the entire network to the winning neurons as training progresses. In addition, the learning rate η changes during training:


(3) $$\eta =\left(\eta^{s\,t\,a\,r\,t}-\eta^{f\,i\,n\,a\,l}\right)\,\left(1-\frac{n_{e\,p\,o\,c\,h}}{n_{t\,o\,t}}\right)+\eta^{f\,i\,n\,a\,l}$$

where *n*_*tot*_ represents the total number of iterations; *n*_*epoch*_ represents the current iteration times.

## 3. SOS algorithm

In nature, some organisms establish symbiotic relationship, which strengthens their ability to adapt to the environment, thereby enhancing their viability. The SOS algorithm (Cheng and Prayogo, 2014) simulates the symbiotic relationships found in nature. Each organism in the ecosystem passes through three phases in the SOS algorithm: mutualism, commensalism, and parasitism. In each phase, the organism is assumed to be in a symbiotic relationship with another random organism. The interactions between the pairs of organisms are used to adjust the fitness value. The result is an optimal solution to the problem. The process is described in the following sections.

### 3.1. Mutualist phase

In nature, the symbiosis between bees and flowers provides a mutual benefit, as both organisms can benefit. The formulae for updating organisms in mutually beneficial symbiosis are as follows:


(4) $$X_{i\,n\,e\,w}=X_{i}+r\,a\,n\,d\,\left(0,1\right)\times \left(X_{b\,e\,s\,t}-M\,u\,t\,u\,a\,l\,\_\,V\,e\,c\,t\,o\,r\times B\,F_{1}\right)$$

(5) $$X_{j\,n\,e\,w}=X_{j}+r\,a\,n\,d\,\left(0,1\right)\times \left(X_{b\,e\,s\,t}-M\,u\,t\,u\,a\,l\,\_\,V\,e\,c\,t\,o\,r\times B\,F_{2}\right)$$

(6) $$M\,u\,t\,u\,a\,l\,\_\,V\,e\,c\,t\,o\,r=\frac{X_{i}+X_{j}}{2}$$

where *X*_*i*_ and *X*_*j*_ represent two of the organisms in the ecosystem. *X*_*best*_ represent the best organism; *Mutual*_*Vector* represents the relationship between two organisms. The benefit factors are *BF*_1_ and *BF*_2_, which have a value of 0 or 1. The unequal benefits obtained by the two parties from the symbiotic relationship are controlled by the benefit factors.

### 3.2. Commensal phase

An example of commensalism in nature is that between a remora and a shark. The remora benefits while the shark is neither harmed nor benefits. This symbiotic relationship is called partiality. The formula for the commensalism phase is


(7) $$X_{i\,n\,e\,w}=X_{i}+r\,a\,n\,d\,\left(-1,1\right)\times \left(X_{b\,e\,s\,t}-X_{j}\right)$$

where *X*_*i*_ is the party that makes a unilateral gain and *X*_*j*_ is the party that is not harmed.

### 3.3. Parasitic phase

In nature, parasitism occurs between mosquitoes and humans. The mosquitoes benefit, whereas the humans are hurt. In this stage, some of the dimensions of *X*_*i*_ are randomly selected and replaced by random values within the search space to form the artificial parasite*Parasite*_*Vector*. In the population randomly selected, we compare the fitness of an individual*X*_*j*_(*j*≠*i*) with *Parasite*_*Vector*, and keep the optimal organism as the new *X*_*j*_.

## 4. Proposed SOS-Kohonen algorithm for intrusion detection

The SOS algorithm is based on the natural phenomenon of symbiosis between various organisms. When a virus intrudes into a system, the relation between the system and the virus can be viewed as a symbiotic relationship between the virus data and the system data.

The initial weights of the Kohonen neural network are optimized with the SOS algorithm. The optimized Kohonen neural network can reduce the length of the error vector between the training sample and the weight vector. This process helps to avoid rigidity during training, which can improve the clustering ability of the Kohonen neural network. After SOS training, the Kohonen neural network identifies subclasses with similar input patterns. Each subclass is used to train a specific radial basis network, which results in a local adjustment of the weights of the radial basis network. This can reduce the training burden of the radial basis network and improve the classification of the sample. For a Kohonen neural network trained by a sample data set, only one neuron in the competing layer is activated. A radial basis network corresponding to the activated winning neurons is used as input. Currently, the only neuron in the output layer is the transient stability index.

The steps in SOS-Kohonen intrusion detection are as follows:

*Step 1*. Initialize the training data set, the number of symbiotic species, and the number of iterations.

*Step 2*. The initial weights *w* are adjusted according to the Euclidean distance between the sample vector and the initial weight in the mutualist phase, commensal phase, and parasitic phase of the SOS algorithm.

*Step 3*. The Kohonen neural network is trained according to the initial weight *w* optimized by the SOS algorithm.

*Step 4*. The distance between the competing layer neuron *j* and the input vector *X* is calculated:


(8) $$d_{j}=\left|\sum_{i=1}^{m}{\left(x_{i}-w_{i\,j}\right)}^{2}\right|,j=1,2,\ldots ,n.$$

*Step 5.* If the minimum distance has been reached, the competing layer neuron *X*, which matches the sample vector *C*, is the output neuron of the optimal matching.

*Step 6*. Adjust the node weight coefficients in node *c* and neighborhood vector *x*:


(9) $$N_{c}\left(t\right)=\{t|find\left(norm\left(pos_{t},pos_{c}\right)<r\right\},j=1,2,\ldots ,n.$$

$$w_{i\,j}=w_{i\,j}+\eta \,\left(X_{i}-w_{i\,j}\right)$$

The positions of neurons *c* and *t* are denoted by *pos*_*c*_ and *pos*_*t*_, respectively. The distance between the two neurons is calculated in terms of *norm*(). η and *r* represent the learning rate and the neighborhood radius, respectively. They decrease linearly as the number of iterations increases.

*Step 7.* If the stopping condition is met stop, otherwise return to step 3.

*Step 8*. Read another test data set.

*Step 9*. Cluster the input test data set according to the trained weight *W*.

*Step 10*. Output the classification result.

Pseudocode corresponding to the steps of SOS-Kohonen intrusion detection is given in Algorithm 1.

**Algorithm 1:** SOS-Kohonen neural network for virus intrusion detection.

**Initialize:** Populate *n* organisms in the ecosystem with random values **Input:** Training data set Calculate the initial weights *w* by summing the points and output nodes of the Kohonen neural network Calculate the fitness of each organism Identify the best organism (*X*_*best*_) in the initial population Define a stopping criterion (either a fixed number of generations/iterations or accuracy) **while (*t* < MaxGeneration**) **for** *i* = 1 to *n* *Mutualist phase* Choose organism *j* randomly other than organism *i* Determine the beneficial factor and mutual vector via Eqs. (6) Modify organisms *X*_*i*_ and *X*_*j*_ based on their mutual relationship via Eqs. (4) and (5) Calculate new weights Evaluate the fitness of the new solution Accept the new solution if the fitness is better *End of mutualist phase* *Commensal phase* Choose organism *j* randomly other than organism *i* Modify organism *X*_*i*_ with the assist of organism *X*_*j*_ via Eq. (7) Calculate new weights Evaluate the fitness of the new solution Accept the new solution if the fitness is better *End of commensal phase* *Parasitic phase* Choose organism *j* randomly other than organism *i* Create a parasite (*Parasite_Vector*) from organism *X*_*i*_ Calculate the fitness of the new organism Kill organism *j* and replace it with the parasite if its fitness is lower than the parasite’s fitness Calculate new weights Evaluate the fitness of the new solution Accept the new solution if the fitness is better *End of parasitism phase* Update the best organism **end for** *t* = *t* + 1 **end while** Calculate the weight of the network according to the individual training weights *w* Read another test data set Cluster the input test data set based on the trained weights **Output:** Classification result

The detection processes of BA-Kohonen, CS-Kohonen, FPA-Kohonen, GWO-Kohonen, and PSO-Kohonen can be imitated by SOS-Kohonen.

## 5. Data preprocessing

In intrusion detection, the network data to be assessed have multiple attributes with inconsistent units of measurement. If such data are used directly for intrusion detection, the accuracy and speed will be reduced. Therefore, the input data are pretreated, that is, normalized. The specific preprocessing method is as follows:

(1) The data are standardized so that the mean of each attribute is 0 and the variance is 1. The attributes of the initial network data are denoted by *x*_*ij*_, and $\bar{x_{j}}$ and *S*_*j*_ represent the mean and variance of the *j*th dimension, respectively. The attributes are standardized as follows:


(10) $$\bar{x_{j}}=\frac{1}{n}\,\sum_{i=1}^{n}x_{i\,j}$$

(11) $$S_{j}=\sqrt{\frac{1}{n-1}\,\sum_{i=1}^{n}{\left(x_{i\,j}-x_{j}\right)}^{2}}$$

The normalized formula for (10) is as follows:


(12) $$x_{i\,j}^{\prime }=\frac{x_{i\,j}-\bar{x_{j}}}{S_{j}},i=1,2,\ldots ,n,j=1,2,\ldots ,m$$

(2) Normalize formula (10) to the range [0, 1] is as follows:


(13) $$x_{i\,j}^{\prime }=\frac{x_{i\,j}-\min\left(x_{i\,j}\right)}{\max\left(x_{i\,j}\right)-\min\left(x_{i\,j}\right)},i=1,2,\ldots ,n,j=1,2,\ldots ,m$$

## 6. Simulation experiments and analysis of results

To verify the effectiveness of SOS-Kohonen in detecting network intrusion by a virus, we ran two sets of tests with the proposed algorithm. The first verified the accuracy of SOS-Kohonen in classifying five types of virus. The second verified the ability of SOS-Kohonen to detect viruses hidden in normal data. The results for SOS-Kohonen were compared with results for Kohonen neural networks combined with one of five commonly used swarm intelligence algorithms: BA, CS, FPA, GWO, and PSO. The relevant parameters for these algorithms were set as follows:

BA: As in Ref. (Xinshe, 2010), r^0^ = 0.5,*A* = 0.5,α = 0.95,γ = 0.05.

CS: As in Ref.(Yang and Deb, 2009), β = 1.5, ρ_0_ = 1.5.

FPA: As in Ref. (Yang, 2012),ρ = 0.8.

GWO: As recommended in Ref. (Mirjalili et al., 2014), $\vec{\alpha }$ = 0 to 2.

PSO: Weight factor ω = 0.6,*c*_1_ = *c*_2_ = 2(Kennedy and Eberhart, 1995), and population size is 15.

SOS: As in Ref. (Cheng and Prayogo, 2014), population size is 15.

### 6.1. Experimental setup

The development environment for this test was MATLAB R2012a. The tests were run on an AMD Athlont (tm) II*4640 processor with 4 GB of memory.

### 6.2. Simulation of virus classification by SOS-Kohonen

**FIGURE 2:**
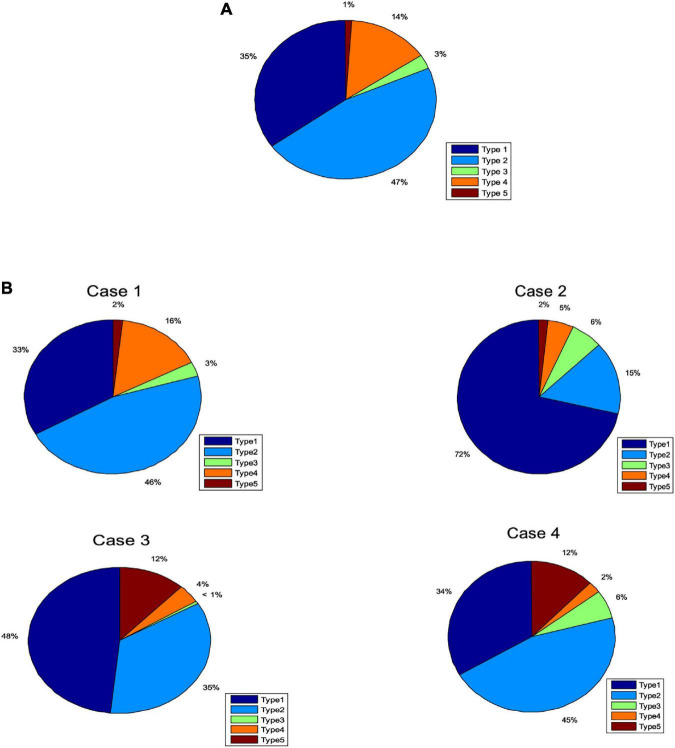
Percentages of each attack type for **(A)** training data set. **(B)** Four randomly selected cases.

In this section, we tested the accuracy of SOS-Kohonen in virus classification. The standard network intrusion test data set contains five categories of virus data. We extracted 4000 training samples, as shown in Table 1 and Figure 2A. Each sample contained a 38-dimensional feature that is used to represent the different attributes of the network intrusion data. Attack type 5 had the fewest training samples and type 2 had the most.

**TABLE 1:** Number of samples for each attack type.

Attack type	Number of samples
1	1,399
2	1,862
3	115
4	580
5	44

Four subsets were randomly selected from the virus intrusion detection data set as test cases. The percentages for the five attack types in the four cases varied (Figure 2B). The number of samples in each case was different, as shown in Table 3.

**TABLE 2:** Statistics for results for the four cases for each algorithm.

Case	Result	Method					
		**BA-Kohonen**	**CS-Kohonen**	**FPA-Kohonen**	**GWO-Kohonen**	**PSO-Kohonen**	**SOS-Kohonen**
1	Best	0.033333	0.06	0.051111	0.04	0.046667	0.033333
	Worst	0.537778	0.191111	0.2	0.602222	0.206667	0.064444
	Mean	0.270444	0.100889	0.14	0.152667	0.117778	0.048889
	Std	0.177392	0.050231	0.065822	0.172365	0.072153	0.008446
2	Best	0.033333	0.017647	0.019608	0.014379	0.01634	0.010458
	Worst	0.219608	0.061438	0.066013	0.799346	0.066013	0.019608
	Mean	0.153529	0.03085	0.045425	0.113725	0.038431	0.015229
	Std	0.071594	0.017234	0.021783	0.242131	0.022362	0.003173
3	Best	0.011494	0.008812	0.010345	0.008812	0.008429	0.006897
	Worst	0.401149	0.033333	0.038697	0.608046	0.038697	0.011494
	Mean	0.242261	0.012912	0.02908	0.08636	0.028467	0.009464
	Std	0.182699	0.007303	0.012269	0.18368	0.012612	0.001666
4	Best	0.026608	0.007895	0.008772	0.004386	0.006433	0.005556
	Worst	0.48655	0.029532	0.029532	0.484795	0.029532	0.008772
	Mean	0.161374	0.015	0.024415	0.057018	0.015058	0.007661
	Std	0.214127	0.009504	0.008512	0.150482	0.009798	0.001101

**TABLE 3:** Comparison of detection rates for the six algorithms.

Case	Attack type					Detection rate					
	**1**	**2**	**3**	**4**	**5**	**BA**	**CS**	**FPA**	**GWO**	**PSO**	**SOS**
1	149	208	14	71	8	97.84%	97.78%	97.62%	97.73%	96.36%	98.53%
2	1094	235	95	78	28	99.07%	99.29%	99.04%	98.93%	99%	99.54%
3	1263	914	17	108	308	87.57%	99.08%	99.56%	99.43%	99.61%	99.73%
4	1146	1556	212	81	425	99.63%	99.66%	99.63%	99.74%	99.69%	99.78%
Average detection rate						96.03%	98.95%	98.96%	98.96%	98.66%	99.4%

For each case, we conducted 10 independent tests using each of the six group intelligence algorithms to determine the weights for the Kohonen neural network. It can be seen that the SOS-Kohonen algorithm has a preference better than BA-Kohonen, CS-Kohonen, FPA-Kohonen, GWO-Kohonen, or PSO-Kohonen, both in terms of optimal value and variance for the accuracy. It also had stronger robustness.

**FIGURE 3:**
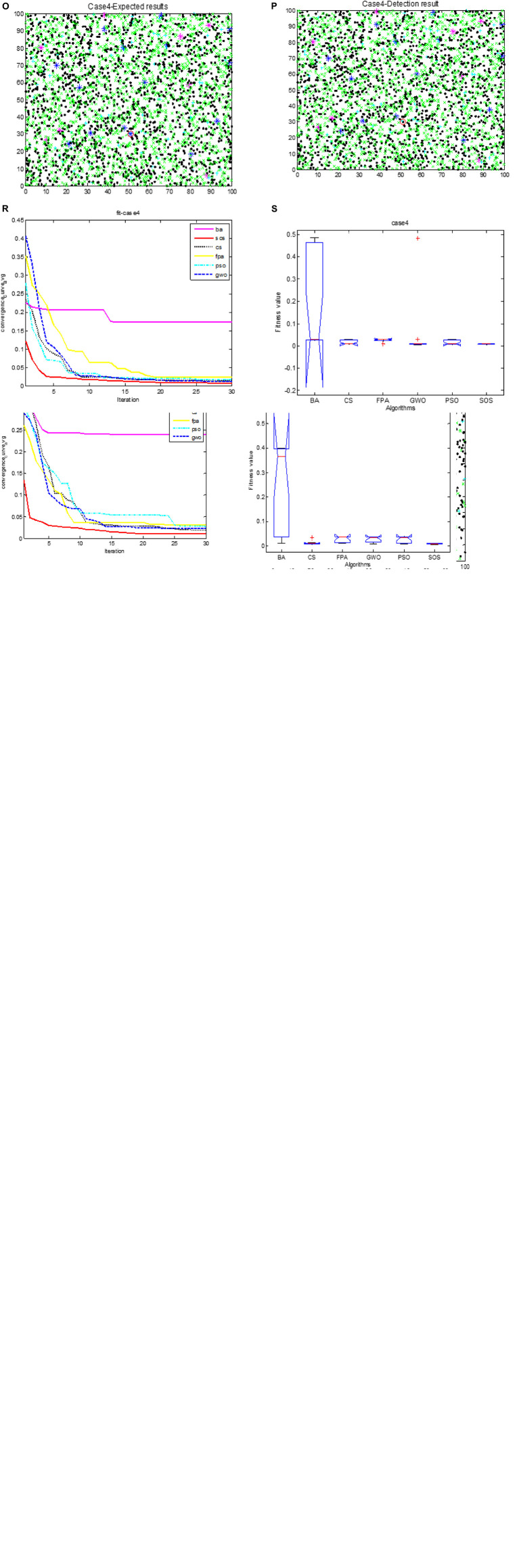
Cases 1- 4 expected classification results are **(C,G,K,O)**; actual classification results are **(D,H,L,P)**; fitness evolution curves are **(E,I,M,R)**; ANOVA test of optimal paths are **(F,J,N,S)**; respectively.

Figures 3C, G, K, O show the expected classification results for cases 1 to 4. Figures 3D, H, L, P show the actual results for these cases for SOS-Kohonen. Due to space constraints, we show the results only for the highest detection rate from the 10 independent runs. The detection rate is defined in Section “6.3. Simulation of virus detection by SOS-Kohonen.” The red circles indicate differences between the actual detection and the expected detection. Figures 3D, L, P have only one error, whereas Figure 3H has five.

Table 3 lists the detection rates for the six algorithms for the four cases. SOS-Kohonen had higher detection rates than the BA-Kohonen, CS-Kohonen, FPA-Kohonen, GWO-Kohonen, or PSO-Kohonen algorithms. It achieved an average detection rate of 99.4% in classifying intrusion data.

Figures 3E, I, M, R illustrate the convergence of the six algorithms. It can be seen that SOS converged fastest and with the highest accuracy. Figures 3F, J, N, S are variance maps for each algorithm. The SOS algorithm had the strongest stability and highest robustness compared to the other algorithms.

### 6.3. Simulation of virus detection by SOS-Kohonen

This section uses the internationally accepted KDDCUP99 (KDD Cup 1999 Data, 1999; MIT Lincoln Laboratory, 2009; Aggarwal and Sharma, 2015) data set to verify the detection performance of SOS-Kohonen. The KDDCUP99 data set was established by the Lincoln Laboratory of the Massachusetts Institute of Technology. The data set was collected using tcpdump from a simulated network environment over 9 weeks. This database has become a benchmark for network intrusion detection and can be used in comprehensive tests of the performance of intrusion detection algorithms. Attacks in the data set include denial of service attacks (DOS), scan attacks (probe), remote user unauthorized access attacks (U2L), and unauthorized use of local super-privilege access attacks (U2R). We apply the internationally accepted detection rate and false alarm rate as evaluation indicators, which are defined as follows (Ganapathy et al., 2012; Lin et al., 2015):


(14) $$\mathrm{Detection}\,\mathrm{rate}=\frac{\mathrm{Number}\,\mathrm{of}\,\mathrm{attack}\,\mathrm{samples}\,\mathrm{found}}{\mathrm{Total}\,\mathrm{number}\,\mathrm{of}\,\mathrm{attacks}}\times 100\%$$

(15) $$\mathrm{False}\,\mathrm{alarm}\,\mathrm{rate}=\frac{\begin{array}{c} \mathrm{Number}\,\mathrm{of}\,\mathrm{correct}\,\mathrm{samples}\\ \mathrm{that}\,\mathrm{were}\,\mathrm{misjudged} \end{array}}{\mathrm{Total}\,\mathrm{number}\,\mathrm{of}\,\mathrm{correct}\,\mathrm{samples}}\times 100\%$$

**FIGURE 4:**
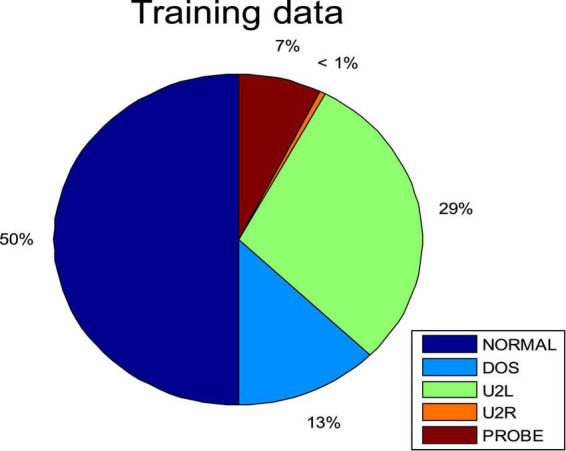
Percentage of each attack type in the training data set.

**FIGURE 5:**
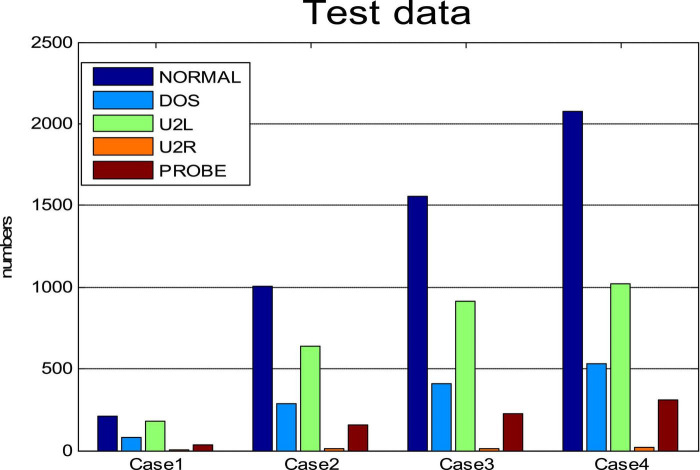
Specific proportions of the test data.

We randomly selected 6,000 samples as training data, including normal data and the four kinds of intrusion data. The percentages of these five types of data are given in Figure 4. Among them, normal samples were the most common and U2R samples the least common. We then randomly selected four subsets from the KDDCUP99 data set as test cases. The number of each attack type for each case are plotted in Figure 5 and listed in Table 4.

**TABLE 4:** Number of samples for each test case for each attack type.

Case	Attack type				
	**Normal**	**DOS**	**U2L**	**U2R**	**Probe**
1	308	78	178	3	33
2	1007	290	636	10	157
3	1558	409	913	15	225
4	2078	529	1024	21	308

In this paper, 10 independent experiments were carried out for each algorithm for the four cases. As can be seen from Table 5, for cases 1, 2, and 3, the SOS-Kohonen algorithm has higher search accuracy than the other algorithms. In case 4, although the optimal value for SOS is slightly worse than that for GWO, the average of the 10 runs was still better than that of the other five algorithms. This shows that the SOS-Kohonen algorithm has a strong search ability and robustness as a whole.

**TABLE 5:** Statistics for accuracy for the four cases for each algorithm.

Case	Result	Method					
		**BA-Kohonen**	**CS-Kohonen**	**FPA-Kohonen**	**GWO-Kohonen**	**PSO-Kohonen**	**SOS-Kohonen**
1	Best	0.106667	0.2	0.198333	0.095	0.151667	0.09
	Worst	0.488333	0.331667	0.375	0.361667	0.365	0.236667
	Mean	0.3475	0.282833	0.2965	0.201667	0.284	0.156333
	Std	0.11302	0.037392	0.056675	0.102524	0.071868	0.065106
2	Best	0.258095	0.239048	0.273333	0.161429	0.142857	0.082857
	Worst	0.560476	0.36381	0.397619	0.690952	0.394762	0.252857
	Mean	0.391762	0.306048	0.319762	0.329905	0.274238	0.166524
	Std	0.085917	0.046231	0.034559	0.177668	0.081504	0.054313
3	Best	0.251923	0.107692	0.298397	0.150321	0.185897	0.11859
	Worst	0.482372	0.379487	0.384295	0.365705	0.380128	0.294231
	Mean	0.361699	0.273526	0.356571	0.257981	0.320897	0.187404
	Std	0.082383	0.081583	0.025715	0.085018	0.065687	0.052848
4	Best Worst Mean Std	0.253382 0.497826 0.389348 0.091809	0.173913 0.339855 0.27657 0.050181	0.251691 0.394444 0.319324 0.049568	0.089372 0.378986 0.232729 0.106131	0.189614 0.419082 0.326715 0.067197	0.104106 0.301449 0.2143 0.059002

**FIGURE 6:**
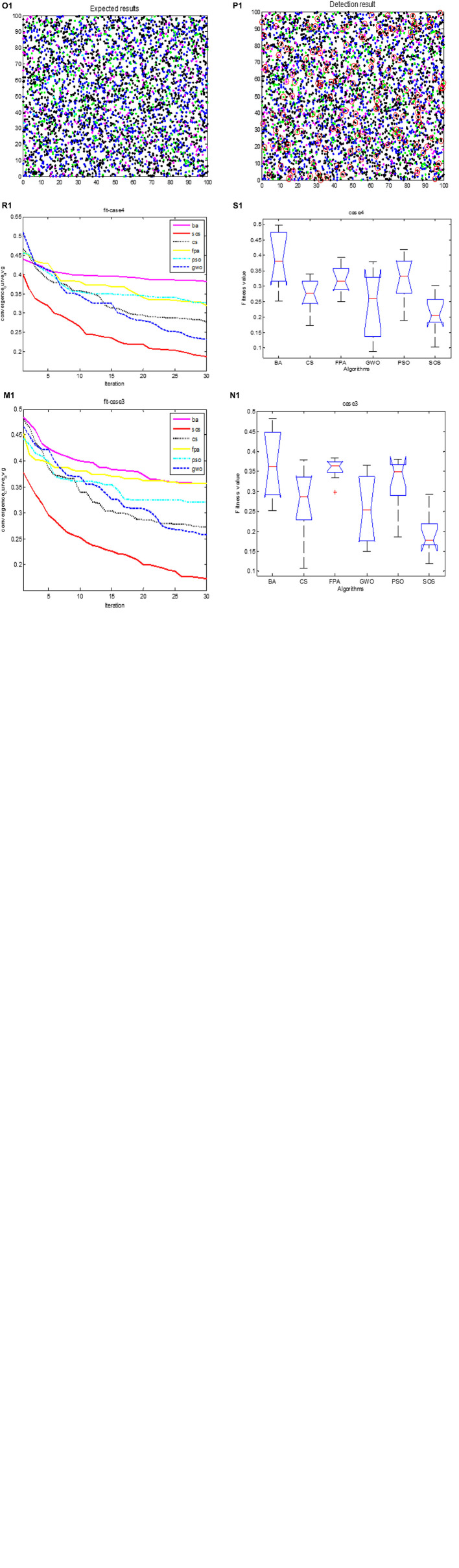
Cases 1–4 expected classification results are **(C_**1**_,G_**1**_,K_**1**_,O_**1**_)**; actual classification results are **(D_**1**_,H_**1**_,L_**1**_,P_**1**_)**; fitness evolution curves are **(E_**1**_,I_**1**_,M_**1**_,R_**1**_)**; ANOVA test of optimal paths are **(F_**1**_,J_**1**_,N_**1**_,S_**1**_)**; respectively.

Figures 6C1, G1, K1, O1 show the expected test results for cases 1 to 4, and Figures 6D1, H1, L1, P1 show the actual test results for SOS-Kohonen. Due to space constraints, we show the results only for the highest detection rate from the 10 independent runs. The normal data are represented as blue dots, and the other colors represent the four types of intrusion data. The red circles indicate differences between the actual detection and the expected detection.

Table 6 shows the average detection rates and average false alarm rates for the six algorithms for the four cases. It can be seen that SOS-Kohonen had a higher detection rate and lower false alarm rate than BA-Kohonen, CS-Kohonen, FPA-Kohonen, GWO-Kohonen, or PSO-Kohonen. The average detection rate of SOS-Kohonen was 94.51%.

**TABLE 6:** Comparison of average detection rate and average false alarm rate for the six algorithms.

Case	Result	Method					
		**BA**	**CS**	**FPA**	**GWO**	**PSO**	**SOS**
1	Detection rate	85.24%	89.69%	90.75%	88.56%	90.55%	95.45%
	False alarm rate	10.33%	7.98%	7.25%	7.97%	7.47%	5.20%
2	Detection rate	84.38%	90.65%	87.58%	87.56%	84.76%	96.26%
	False alarm rate	12.34%	7.94%	9.91%	8.76%	11.25%	4.67%
3	Detection rate	91.59%	90.13%	83.69%	89.28%	80.54%	94.90%
	False alarm rate	8.11%	7.47%	10.82%	8.32%	12.17%	5.44%
4	Detection rate	85.42%	86.14%	80.45%	84.19%	90.39%	91.43%
	False alarm rate	9.43%	10.50%	11.77%	11.69%	7.07%	6.79%
Average detection rate		86.66%	89.16%	85.62%	87.40%	86.56%	94.51%

Figures 6E1, I1, M1, R1 show the convergence of the six algorithms. The convergence speed and accuracy of SOS-Kohonen were better than those of the other algorithms. Figures 6F1, J1, N1, S1 show the variance of each algorithm. For each case, the SOS-Kohonen algorithm had the second best variance ranked second but the highest search accuracy. Overall, the SOS-Kohonen algorithm performed better than the other algorithms.

### 6.4. *p*-values from the Wilcoxon rank-sum test

Next, we ran the Wilcoxon rank-sum test (Derrac et al., 2011; Gibbons and Chakraborti, 2011; Hollander et al., 2013) for SOS-Kohonen and the other five algorithms. We chose *p* = 0.05 as the level of significance. Table 7 shows the *p*-values for the four classification cases described in Section “6.2. Simulation of virus classification by SOS-Kohonen.” Table 8 shows the *p*-values for the four detection cases described in Section “6.3. Simulation of virus detection by SOS-Kohonen”

**TABLE 7:** *p*-values from the Wilcoxon rank-sum test for the four classification cases.

Algorithm	Case 1	Case 2	Case 3	Case 4
SOS vs. BA	0.0079	1.7462 × 10^–4^	2.3697 × 10^–4^	1.6589 × 10^–4^
SOS vs. CS	4.7751 × 10^–4^	0.0021	0.0608	0.0051
SOS vs. FPA	4.8226 × 10^–4^	3.8732 × 10^–4^	7.2031 × 10^–4^	2.5197 × 10^–4^
SOS vs. GWO	0.0404	0.0441	0.0012	0.9696
SOS vs. PSO	0.0062	0.0013	0.0023	0.1267

**TABLE 8:** *p*-values from the Wilcoxon rank-sum test for the four detection cases.

Algorithm	Case 1	Case 2	Case 3	Case 4
SOS vs. BA	0.0022	1.8165 × 10^–4^	4.3745 × 10^–4^	7.6502 × 10^–4^
SOS vs. CS	4.3964 × 10^–4^	3.2643 × 10^–4^	0.0172	0.0312
SOS vs. FPA	5.8006 × 10^–4^	1.8063 × 10^–4^	1.8165 × 10^–4^	0.0017
SOS vs. GWO	0.5452	0.0058	0.0639	0.6232
SOS vs. PSO	0.0028	0.0073	0.0010	0.0036

In Table 7, for case 3, the *p*-value for SOS vs CS is greater than 0.05. For case 4, the *p*-values for SOS vs GWO and SOS vs PSO are greater than 0.05. All other *p*-values are less than 0.05. In Table 8, the only *p*-values greater than 0.05 are for SOS vs GWO for cases 1, 3, and 4. Thus, for most of the eight tests cases, the differences between SOS and the other algorithms were statistically significant and not due to chance.

### 6.5. Analysis of results

In this paper, six common swarm intelligence algorithms were combined with Kohonen neural network and used to simulate the intrusion detection of network viruses. We ran two sets of tests. In Section “6.2. Simulation of virus classification by SOS-Kohonen,” we tested the classification accuracy of the algorithms. Figures 3C—S show the classification results, convergence, and variance maps for the four test cases. Table 2 lists the classification accuracy of the six swarm intelligence algorithms.

In Section “6.3. Simulation of virus detection by SOS-Kohonen,” we assessed the detection rate and false alarm rate for normal data and four attack types. Figures 6C1—S1 show the classification results, convergence, and variance maps for the four test cases. Table 6 compares the average detection rates and false alarm rates for the six algorithms.

In Section “6.4. *p*-values from the Wilcoxon rank-sum test,” we ran the Wilcoxon rank-sum test. For most of the eight tests cases, the differences between SOS and the other algorithms were statistically significant and not due to chance. Thus, the SOS-Kohonen algorithm is more effective than the other five swarm intelligence algorithms in detecting network intrusion by a virus.

## 7. Conclusions and future work

With the continuous development and popularization of the Internet, there is much more convenient access to network resources. However, this has led to a continuous increase in security problems due to virus intrusion. In this paper, we combined a swarm intelligence algorithm with a neural network to detect network intrusion by a virus. Our approach is described in detail, and it was tested with the international KDDCUP99 intrusion data set, which verified its effectiveness. Moreover, this is also a new method for detecting network intrusion by a virus. With the rapid development of cloud computing and big data, our future work will consider the application of SOS- Kohonen to heterogeneous distributed systems.

## Data availability statement

The original contributions presented in this study are included in the article/supplementary material, further inquiries can be directed to the corresponding authors.

## Author contributions

GZ: investigation, experiments, and writing the draft. FM: algorithm design. ZT: algorithm analysis. YZ: supervision, reviewing, and editing the manuscript. QL: reviewing and editing the manuscript. All authors contributed to the article and approved the submitted version.
